# Controlled venospasm-assisted foam sclerotherapy combined with high ligation—a novel minimally invasive approach for primary great saphenous vein varicosities

**DOI:** 10.3389/fsurg.2025.1730329

**Published:** 2026-01-20

**Authors:** Chen Ya, Liu Zechao, Zhu Xuchang, Chen Boyu, Liu Zhengli, Kong Jie

**Affiliations:** 1 Department of Interventional Radiology, Jingzhou Hospital Affiliated to Yangtze University, Jingzhou, Hubei, China; 2 Department of Interventional Radiology, Lianshui County People’s Hospital, Lianshui, Jiangsu, China; 3 Department of Vascular Surgery, Lianshui County People’s Hospital, Lianshui, Jiangsu, China; 4 Department of Interventional Radiology, Nanjing First Hospital, Nanjing Medical University, Nanjing, Jiangsu, China

**Keywords:** controlled venospasm, foam sclerotherapy, great saphenous vein, minimally invasive, varicosities

## Abstract

**Purpose:**

This study aimed to evaluate the efficacy and safety of Controlled Venospasm-Assisted Foam Sclerotherapy (CVAFS) combined with high ligation (HL) for treating primary great saphenous vein (GSV) varicosities.

**Materials and methods:**

A retrospective cohort of 127 patients with primary GSV varicosities underwent CVAFS with high ligation between 1 Jan 2023 and 1 October 2023. Venospasm was induced by rotational mechanical stimulation of the catheter combined with external compression, resulting in a transient reduction of vessel diameter by 50%–70%, followed by DSA (Digital subtraction angiography)-guided foam injection (1:4 liquid-to-gas ratio). Technical success was defined as complete procedural execution under imaging guidance. Primary endpoints included 1-year GSV occlusion rate (assessed by duplex ultrasound) and reduction in Venous Clinical Severity Score (VCSS). Complications were recorded and managed conservatively.

**Results:**

Technical success was achieved in 100% of limbs (145/145). Among 127 enrolled patients, 109 patients (125 limbs) completed the 12-month follow-up, yielding a follow-up rate of 85.8% (109/127). At 12 months, 93.6% of great saphenous veins (117/125 limbs) maintained complete occlusion. The Venous Clinical Severity Score (VCSS) significantly decreased from 6.18 ± 3.90 preoperatively to 0.86 ± 0.90 postoperatively (*V* = 7,875, *p* < 0.001). Thrombophlebitis observed in 9.6% of limbs (12/125), all cases resolved spontaneously within 2 weeks with conservative management (warm compression and NSAIDs). Saphenous Junction Pain occurred in 32.8% of limbs (41/125), with complete resolution within 2 weeks without intervention. No deep venous thrombosis (DVT), pulmonary embolism, skin necrosis, or neurological injuries were documented.

**Conclusion:**

CVAFS leverages controlled venospasm to enhance foam-endothelium contact, significantly improving occlusion rates and symptom relief with acceptable safety. This approach offers a promising minimally invasive alternative for GSV varicosities.

## 1 Introduction

The focus of this investigation is primary great saphenous vein (GSV) varicosities, a manifestation of chronic venous insufficiency affecting 20%–40% of Western populations (1, 2). Pathologically characterized by venous wall dilation and valvular incompetence, this condition progresses through CEAP clinical stages C2-C6, manifesting as pain, edema, skin hyperpigmentation, and ulceration (3). Beyond imposing substantial patient morbidity and quality-of-life impairment, it accounts for approximately 2% of national healthcare expenditures in developed countries (4).

Current guidelines recommend thermal ablation, sclerotherapy, or surgical stripping for GSV management (1, 2). Foam sclerotherapy—through detergent-induced endothelial damage leading to fibrotic occlusion—represents a minimally invasive option with reported 3-year closure rates of 70%–85% (5). However, hemodynamic limitations constrain its efficacy: in large-diameter (>8 mm) GSVs with high flow velocities (>10 cm/s), sclerosant dilution reduces endothelial contact time, diminishing foam stability and biological effectiveness (5–7). Computational fluid dynamics models indicate turbulence reduces therapeutic agent residence time by 40%–60% compared to low-flow states (8–10).

The induction of transient venospasm has been proposed as a potential strategy to treat vein varicosities. Transient vasoconstriction reduces luminal diameter and blood velocity, thereby prolonging contact between the sclerosant and the endothelium (5, 11, 12). To translate this physiological principle into a viable clinical application while addressing the limitations of existing therapies, we developed a hybrid approach: Controlled Venous Spasm-Assisted Foam Sclerotherapy (CVAFS) combined with High Ligation (HL). Controlled venous spasm was achieved through endovenous mechanical stimulation via a 4F vertebral catheter rotated and pulled back within the venous lumen, augmented by targeted external manual compression to induce transient vasoconstriction.

This retrospective cohort study evaluated the efficacy of CVAFS + HL, a technique combining high ligation for proximal flow interruption and pharmacologically-induced spasm for distal flow modulation, in 127 patients with primary great saphenous vein (GSV) varicosities (CEAP C2-C4). The primary objectives were to assess the 12-month anatomical closure rate of the GSV trunk, particularly in the thigh segment, complication rates (including thrombophlebitis and neurological injury), and improvement in Venous Clinical Severity Score (VCSS).

## 2 Materials and methods

### 2.1 Study design and treatment strategies

Approval for this retrospective cohort study was obtained from the Ethics Committee of our center. Utilizing the electronic medical record system, two independent reviewers identified individuals with primary great saphenous vein (GSV) varicosities. Between January 1, 2023, and October 1, 2023, our center conducted 145 CVAFS + HL procedures on 127 patients, including cases with bilateral interventions. Demographic and clinical characteristics, such as age, gender, and comorbid conditions (e.g., hypertension and diabetes), were documented. All participants underwent standardized CEAP classification and Venous Clinical Severity Score (VCSS) assessments during initial evaluation.

CVAFS, incorporating high ligation for proximal flow interruption, was performed only on patients meeting predefined inclusion criteria: 1) sapheno-femoral reflux duration exceeding 1 s, 2) CEAP clinical stage ≥2, 3) patent deep veins in the lower limbs, and 4) GSV diameter ≥5 mm. Exclusion criteria comprised: 1) contraindications to surgical procedures based on clinical status, 2) contraindications to foam sclerotherapy, 3) pregnancy, 4) history of DVT and 5) thrombophilia as summarized in Table 1.

**Table 1:** Selection criteria.

Inclusion and Exclusion criteria
**Inclusion criteria:**
Reflux time at saphenous-femoral valve > 1s
CEAP clinical grade ≥2
The lower extremity deep vein was patentcy
**Exclusion criteria:**
Clinical state not suitable for surgery
Patients with contraindication to foam sclerosant treatment

### 2.2 Interventional procedure

**Figure 1:**
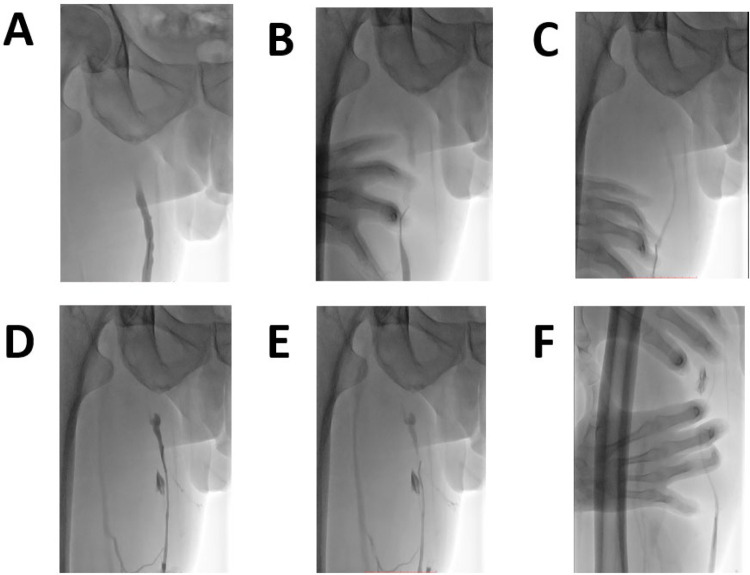
Procedural steps of controlled venous spasm-assisted foam sclerotherapy with high ligation (CVAFS + HL) under venographic guidance. **(A)** Proximal great saphenous vein (GSV) occlusion confirmed by manual contrast injection via catheter (white arrow indicates occlusion site). **(B)** Venographic validation of >50% reduction in venous diameter post-venospasm induction. **(C,D)** Mechanical venospasm induction: A 4-Fr catheter is rotated and retracted within the GSV lumen while external cutaneous compression is applied. **(E)** Injection of polidocanol foam (1% concentration) prepared via Tessari method (1:4 liquid-to-gas ratio); foam dispersion pattern shown by radiolucent bubbles. **(F)** Concurrent manual compression of perforating veins to prevent foam migration into deep veins; dashed arrow denotes direction of compression force.

Patients were positioned supine with local anesthesia (1% lidocaine) administered at the puncture site. Following sterile preparation, ultrasound-guided puncture targeted the great saphenous vein (GSV) trunk at the medial knee joint, facilitating insertion of a 5-Fr introducer sheath. Manual contrast injection subsequently localized the sapheno-femoral junction (SFJ), enabling high ligation 1.0 cm distal to the SFJ using non-absorbable sutures as described before (13). Proximal GSV occlusion was confirmed via manual contrast injection through the catheter (Figure 1A). A 4-Fr catheter was advanced into the GSV lumen, and mechanical venospasm induction ensued through combined catheter rotation/retraction and external cutaneous compression (Figures 1B,C), achieving >50% venous diameter reduction as verified by contrast imaging (Figure 1D). Mechanical venospasm was induced by rotating the 4F vertebral catheter approximately 5–7 times per segment while retracting it at a rate of ∼1 cm/s. Concurrently, targeted external manual compression was applied to achieve a transient reduction in venous diameter by 50%–70%, as confirmed by contrast venography. To minimize inter-operator variability, all procedures were performed by two experienced interventional radiologists who were trained together using a standardized protocol. Sclerosing foam—prepared immediately using the Tessari method (1:4 liquid-to-gas)—was injected through the catheter, with a total administered volume of approximately 5 mL (Figure 1E), while concurrent manual compression of perforating veins prevented foam migration into the deep venous system (Figure 1F). Below-knee varicosities received adjunctive DSA(Digital subtraction angiography)-guided foam injection followed by massage for dispersion uniformity, while ambulatory phlebectomy addressed tributaries >4 mm or clustered varicosities under local anesthesia.

Postprocedural management initiated eccentric compression bandaging across the limb immediately. Patients commenced ambulation 6 h postoperatively, walking 15 min every 2 h. Elastic bandages were maintained for 72 h before transitioning to daily graduated compression stockings (20–30 mmHg) worn for ≥3 months. Duplex ultrasound assessments at 12 months were performed by two sonographers who were blinded to the procedural details, using a standardized imaging protocol to ensure consistency. Complete occlusion was defined as the absence of flow signal on Doppler imaging and non-compressibility of the vein lumen on B-mode imaging. Venous Clinical Severity Score (VCSS) was concurrently quantified to evaluate symptom improvement.

### 2.3 Statistical analysis

Statistical analyses were conducted using R software (version 3.6.2; R Foundation for Statistical Computing). Continuous variables are presented as mean ± standard deviation (SD), while categorical data are summarized as frequencies and percentages [*n* (%)]. Independent samples t-tests were applied to compare normally distributed continuous variables between groups, with homogeneity of variances confirmed by Levene's test. Chi-square tests (or Fisher's exact tests for expected cell counts <5) assessed associations between categorical variables. Wilcoxon rank-sum tests analyzed non-normally distributed continuous or ordinal data. A two-sided *P*-value <0.05 defined statistical significance (14).

## 3 Results

Between January and October 2023, 127 patients underwent controlled venospasm-assisted foam sclerotherapy (CVAFS) combined with high ligation. The cohort had a mean age of 60.2 ± 9.3 years, comprising 62 males and 65 females. Comorbidities included diabetes mellitus (*n* = 34), hypertension (*n* = 53), and other cardiovascular conditions (lacunar infarction or coronary artery disease, *n* = 13) (Table 2).

**Table 2:** Demographic characteristics.

Parameter	127 patients (145 legs)
Age	
Mean (SD)	60.2 (9.3)
Gender	
Male	62 (48.8%)
Hypertension	
Yes	53 (41.7%)
Diabete	
Yes	34 (26.8%)
Other	
Yes	13 (10.2%)
CEAP clinical grade	
C_2_	104 (71.8%)
C_3_	19 (13.1%)
C_4_	16 (11.0%)
C_5_	6 (4.1%)
C_6_	0 (0%)

**Figure 2:**
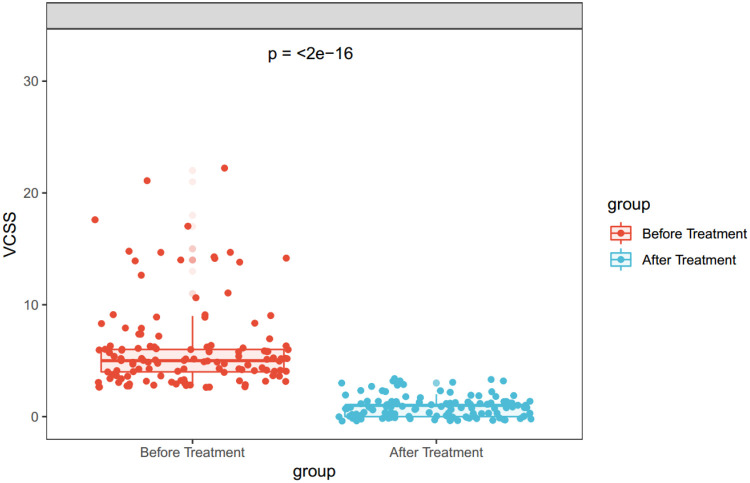
Preoperative versus 12-month postoperative venous clinical severity score (VCSS) comparison. Significant reduction in venous disease severity after CVAFS + HL. Boxplots display median scores (preoperative: 6.18 ± 3.90; 12-month: 0.86 ± 0.90) with interquartile ranges. The median postoperative VCSS decreased by 86.1% relative to baseline (*p* < 0.001, Wilcoxon signed-rank test; *V* = 7,875).

A total of 145 affected limbs were treated, with CEAP clinical classifications distributed as follows: C2 (*n* = 104), C3 (*n* = 19), C4 (*n* = 16), and C5 (*n* = 6) (Table 2). All procedures achieved technical success (145/145 limbs, 100%). Among 127 patients, 109 (85.8%) completed the 12-month follow-up, encompassing 125 treated limbs. Ultrasound assessment confirmed sustained great saphenous vein (GSV) occlusion in 117 limbs (93.6%, 117/125, 95% CI: 89.3%–97.9%), with no cases of clinical recurrence observed. The Venous Clinical Severity Score (VCSS) demonstrated significant improvement from a preoperative baseline of 6.18 ± 3.90 to 0.86 ± 0.90 at 12 months (*p* < 0.001, Wilcoxon signed-rank test; V = 7,875) (Figure 2).

Postprocedural complications included saphenous junction pain (*n* = 41, 32.8%) and thrombophlebitis (*n* = 12, 9.6%), all resolving within two weeks without intervention. No instances of deep venous thrombosis or other severe complications occurred during follow-up.

We compared our results with previous studies to evaluate the performance of CVAFS + HL relative to established treatments for great saphenous vein (GSV) incompetence, as summarized in Table 3. The results demonstrate that CVAFS + HL achieved a technical success rate of 100% (145/145 limbs) within 6 weeks, with a 1-year occlusion rate of 93.6% (117/125 limbs). In comparison, ultrasound-guided foam sclerotherapy (UGFS) showed lower long-term efficacy, with occlusion rates of 23%–33% at 5 years, while endovenous laser ablation (EVLA) and mechanochemical ablation (MOCA) exhibited 1-year occlusion rates of approximately 88% and 80%–82%, respectively (22–24). Surgery (high ligation and stripping, HL/S) had a 5-year occlusion rate of 83%. The complication profile for CVAFS + HL included thrombophlebitis (9.6%) and saphenous junction pain (32.8%), with no instances of deep vein thrombosis (DVT), pulmonary embolism (PE), or skin necrosis. This contrasts with UGFS, which had higher rates of hyperpigmentation (10%–15%) and superficial vein thrombosis (SVT; 5.9–13.7%), and surgery, which was associated with nerve injury (11.3%). Quality of life improvement, measured by the Venous Clinical Severity Score (VCSS), showed a significant reduction from 6.18 ± 3.90 to 0.86 ± 0.90 (86.1% improvement, *p* < 0.001) for CVAFS + HL, comparable to the significant improvements seen with EVLA and MOCA. CVAFS + HL required only local anesthesia with tumescence, allowed outpatient procedures, and facilitated rapid recovery (ambulation within 6 h, return to normal activities in 1–2 days), outperforming surgery which typically necessitated general/regional anesthesia and longer recovery (2–3 weeks). Cost-effectiveness was a key advantage of CVAFS + HL due to the absence of proprietary devices, whereas EVLA and MOCA involved higher costs. CEAP class suitability for CVAFS + HL was confirmed for C2–C4 cases, aligning with guidelines for first-line treatments like EVLA. Long-term data for CVAFS + HL are currently limited to 12 months, whereas other modalities have 3- to 5-year follow-up data available. Overall, these results contextualize CVAFS + HL as a competitive alternative to existing therapies, with efficacy and safety profiles that support its use in clinical practice.

**Table 3:** Comparative analysis of CVAFS + HL versus established treatments for GSV incompetence.

Parameter	CVAFS + HL	UGFS (ESVS 2022 Guidelines)	MOCA (ESVS 2022 Guidelines)	EVLA (ESVS 2022 Guidelines)	Surgery/HL/S (ESVS 2022 Guidelines)
Technical Success (≤6 weeks)	100% (145/145 limbs)	64–75% at 1–3 years	86.5% at 3 years	92% (meta-analysis of 28 RCTs)	83% at 5 years
1-Year Occlusion Rate	93.6% (117/125 limbs)	23–33% at 5 years	80–82% at 3 years	88% at 5 years	83% at 5 years
Complication Profile	Thrombophlebitis: 9.6% Saphenous junction pain: 32.8% No DVT/PE/skin necrosis	Hyperpigmentation: 10–15% SVT: 5.9–13.7% Neurological events: <1%	Induration: 12–18% SVT: 2–13% DVT: 0%–1%	EHIT: 1.7% DVT: 0.3% Phlebitis: Common	Haematoma: 4.8% Wound infection: 1.9% Nerve injury: 11.3%
Quality of Life Improvement	VCSS: 6.18 ± 3.90 to 0.86 ± 0.90 (86.1% improvement, *p* < 0.001)	Improves but less than thermal ablation	Comparable to thermal ablation	Significant improvement in all QoL measures	Significant improvement comparable to EVTA
Anesthesia Requirement	Local anesthesia + tumescence	None required	Local anesthesia	Tumescent anesthesia	General/regional anesthesia
Procedure Setting	Outpatient	Outpatient	Outpatient	Outpatient/Ambulatory	Operating room
Recovery Time	Ambulation after 6 h Return to normal activities: 1–2 days	Immediate ambulation Minimal downtime	Quick recovery Early return to work	1–3 days to normal activities	2–3 weeks recovery Longer return to work
Cost Considerations	Cost-effective (no proprietary devices)	Low cost	Moderate (proprietary device)	High (laser equipment)	High (operating room costs)
CEAP Class suitability	C2-C4	Preferred for veins <6 mm diameter	Suitable for various diameters	First-line for most cases	When EVTA not available
Long-term Data	12-month follow-up	5-year data available showing lower efficacy	3-year data available	5-year + data robust	5-year + data available

## 4 Discussion

The global burden of great saphenous vein (GSV) varicosities remains substantial, with an estimated 30% prevalence among adults imposing significant healthcare costs (4, 15). While established modalities like surgical ligation, endothermal ablation (EVLA/RFA), and ultrasound-guided foam sclerotherapy (UGFS) offer therapeutic options, each carries inherent limitations. Foam sclerotherapy—despite its minimally invasive appeal—demonstrates reduced efficacy in larger-diameter veins (>6 mm), where diminished foam-fill ratios and accelerated blood flow compromise endothelial contact time and promote sclerosant dilution (1, 2, 9). This pathophysiological limitation is corroborated by mechanistic studies highlighting the inverse relationship between venous diameter and sclerotherapy success rates (4, 16, 17).

In addressing this challenge, mechanochemical ablation (MOCA) emerged as an innovative approach combining rotational mechanical endothelial injury with liquid sclerosant infusion (18, 19). Contemporary evidence confirms MOCA's short-term efficacy, achieving 88.2% 1-year anatomical occlusion and 93% clinical improvement (19). Its non-thermal nature significantly reduces postoperative pain (mean VAS: 4.8–19.3 mm vs. 18.6–34.5 mm for RFA/EVLA) and accelerates return to normal activities (median 1 day) (19, 20). Nevertheless, MOCA exhibits declining long-term efficacy—3-year occlusion rates drop to 80%–82%, with veins >7 mm demonstrating substantially higher recanalization risk (20). Additionally, a significant advantage of the CVAFS + HL technique is its potential for improved cost-effectiveness compared to MOCA, which relies on proprietary devices such as the ClariVein® catheter (20). Published studies have reported the cost of the single-use ClariVein® device to be approximately $500 - $700. In contrast, the CVAFS + HL technique induces venospasm using a conventional and reusable 4F vertebral catheter, which represents a minimal and recurrent cost. By eliminating the need for a dedicated, single-use device, CVAFS + HL substantially reduces the per-procedure material cost, potentially enhancing its viability and adoption in healthcare settings with limited resources.

Our study introduces Controlled Venospasm-Assisted Foam Sclerotherapy with High Ligation (CVAFS + HL) as a strategically optimized alternative. This technique replicates MOCA's mechanical endothelial stimulation through controlled manual catheter rotation within the GSV lumen, intentionally inducing localized venospasm. Crucially, CVAFS substitutes liquid agents with foam sclerotherapy while incorporating proximal high ligation (HL) to mitigate reflux and augment closure. The synergistic mechanism operates through:1, Mechanical venospasm reducing venous diameter by >40%, enhancing foam-wall contact; 2, Endothelial microtrauma increasing sclerosant absorption; 3, HL eliminating saphenofemoral reflux, reducing hemodynamic stress on treated segments.

Our results demonstrate that Controlled Venospasm-Assisted Foam Sclerotherapy with High Ligation (CVAFS + HL) achieves clinical outcomes comparable to mechanochemical ablation (MOCA) while offering distinct advantages. Regarding safety equivalence, no deep vein thrombosis, nerve injuries, or clinically significant hematomas occurred. Minor complications, such as transient thrombophlebitis (≤9%), resolved with conservative management, thereby aligning with MOCA's established safety profile. Notably, saphenous junction pain is a recognized and typically transient effect attributable to the high ligation component, and its spontaneous resolution in all our cases within two weeks aligns with this expectation. In terms of technical efficacy, 1-year occlusion rates and symptom improvement (quantified by VCSS reduction) paralleled MOCA benchmarks, while the addition of high ligation (HL) specifically mitigated proximal recurrence risks inherent in pure endovenous techniques. Concerning economic superiority, eliminating proprietary devices like ClariVein® substantially reduced per-procedure costs, enhancing viability in resource-constrained settings. These collective findings affirm CVAFS + HL as a cost-effective refinement of mechanochemical principles. The manual catheter rotation maneuver proved exceptionally safe, with no perforations occurring despite aggressive manipulation—a result attributable to the flexible catheter tip design. Furthermore, integrating HL counteracts the weakness of ultrasound-guided foam sclerotherapy (UGFS) in managing junctional reflux, synergistically improving long-term durability.

Despite these promising outcomes, this retrospective single-center analysis carries inherent limitations, including potential selection bias and unmeasured confounders, which are intrinsic to its non-randomized, observational design (21). Furthermore, the 12-month follow-up period, while adequate for initial efficacy and safety assessment, is insufficient to evaluate long-term durability and recurrence rates, which often manifest beyond 2–5 years. The study also did not formally analyze the learning curve associated with CVAFS + HL; however, anecdotal observation suggests that operator proficiency, reflected in procedural time and consistency, improved with cumulative experience, a factor that should be quantitatively assessed in future prospective studies to inform training requirements. Although the abstract mentions potential cost-effectiveness, a formal economic analysis comparing this hybrid technique to established therapies like EVLA or UGFS was beyond the scope of this initial investigation and remains a critical area for future research. Therefore, future multi-center, randomized controlled trials with longer follow-up, integrated learning curve assessments, and comprehensive cost-effectiveness analyses are essential to validate these findings and thoroughly evaluate the technique's broader applicability and economic value. Specific priority areas for such research include investigating diameter-dependent efficacy—specifically whether CVAFS + HL overcomes MOCA's known diminished efficacy in veins exceeding 7 mm—assessing long-term outcomes such as 3- to 5-year occlusion durability and retreatment rates, and exploring optimal foam formulation parameters, including ideal sclerosant type, concentration, and foam stability characteristics.

## 5 Conclusions

CVAFS + HL represents a technically feasible, economically advantageous evolution in minimally invasive varicose vein management. By integrating targeted mechanical venospasm induction with foam sclerotherapy and high ligation, it addresses critical limitations of standalone UGFS while circumventing MOCA's cost barriers. Our results establish its short-term safety and efficacy equivalence to established techniques. Future prospective studies should prioritize long-term outcomes and standardized protocols to solidify its role in evidence-based therapeutic algorithms.

## Data Availability

The original contributions presented in the study are included in the article/Supplementary Material, further inquiries can be directed to the corresponding author.
